# Repair of Ventricular Septal Defect in Children with *TAB2* Gene Anomalies and Associated Cardiomyopathy

**DOI:** 10.70352/scrj.cr.26-0033

**Published:** 2026-06-17

**Authors:** Shinya Ugaki, Shota Ogura, Kazuma Tsuno, Toshikazu Shimizu, Shio Masuda, Ryo Mafune, Kodai Momoki, Sadataka Kawachi, Izumi Hamaya, Kenji Hoshino, Koji Nomura

**Affiliations:** 1 Department of Cardiovascular Surgery, Saitama Children’s Medical Center, Saitama, Saitama, Japan; 2 Department of Cardiology, Saitama Children’s Medical Center, Saitama, Saitama, Japan

**Keywords:** *TAB2*, ventricular septal defect, cardiomyopathy, children

## Abstract

**INTRODUCTION:**

*TAB2* gene abnormalities are known to cause congenital heart disease, cardiomyopathy, and extracardiac malformations. However, there is a paucity of reports describing cardiac surgery complicated by cardiomyopathy and *TAB2* gene anomalies. We present 2 cases of successful closure of ventricular septal defects (VSDs) with a dilated left ventricle and impaired cardiac function accompanied by *TAB2* gene anomalies.

**CASE PRESENTATION:**

Patient 1, a 7-month-old boy with Klinefelter syndrome and a TAB2 variant, presented with a VSD and extracardiac features. He developed severe congestive heart failure and pulmonary hypertension with impaired biventricular function. Despite medical management, his condition worsened, necessitating surgical patch closure. Postoperatively, he required intensive support, including nitric oxide and inotropes, for low cardiac output and persistent pulmonary hypertension. He was successfully extubated on day 7. Two years after surgery, the patient remains in New York Heart Association (NYHA) functional class I with improved biventricular function. Patient 2, a 1-year-4-month-old boy with a TAB2 gene deletion (6q24.3–q25.1), presented with a VSD, cardiomyopathy, and dysmorphic features. Due to decreased biventricular function, treatment with carvedilol and enalapril maleate was initiated at 3 months of age and his cardiac function gradually improved by 1 year of age. Although surgical patch closure of the VSD was performed, postoperative biventricular failure necessitated a 7-day course of extracorporeal membrane oxygenation. His recovery was further complicated by *Pseudomonas aeruginosa* mediastinitis, requiring mediastinal irrigation. He was eventually extubated 45 days postoperatively and resumed medical therapy. Fifteen months after surgery, the patient remains in NYHA functional class I with significantly improved biventricular function.

**CONCLUSIONS:**

Successful VSD repair was achieved in 2 children with dilated ventricles and impaired cardiac function associated with *TAB2* gene abnormalities. Although continued long-term monitoring is necessary, VSD repair may improve clinical outcomes in patients with *TAB2* gene abnormalities complicated by cardiomyopathy.

## Abbreviations

CHD
: congenital heart defect
ECMO
: extracorporeal membrane oxygenation
EF
: ejection fraction
LV
: left ventricle
MAP3K7
: mitogen-activated protein kinase kinase kinase 7
RV
: right ventricle
TGF
: transforming growth factor
VSD
: ventricular septal defect

## INTRODUCTION

*TAB2* is a gene located on chromosome 6q25.1 that encodes TGF-beta-activated kinase 1 and MAP3K7-binding protein 2.^1–4)^ *TAB2* plays a key role in the development of the heart; its haploinsufficiency of *TAB2* is associated with CHDs and cardiomyopathy.^1–5)^ Furthermore, it is involved in extracardiac manifestations, including short stature, facial dysmorphism, connective tissue abnormalities, and varying degrees of developmental delay.^1–5)^ Although the term “*TAB2*-related syndrome” has been proposed, it is currently still classified as a cause of non-syndromic CHD according to Online Mendelian Inheritance in Man (OMIM#614980).^2)^

Most children with *TAB2* gene anomalies present with CHD, such as valvular disease, LV outflow obstruction, atrial septal defect, and VSD. Many *TAB2*-related CHD cases are asymptomatic, and the existing literature has primarily focused on collecting clinical manifestations of *TAB2* abnormalities. Consequently, there is a paucity of reports describing cardiac surgery for CHD complicated by cardiomyopathy in this population.^1–5)^ We present 2 cases of successful VSD closure in children with *TAB2* abnormalities who presented with dilated LV and impaired cardiac function.

## CASE PRESENTATION

### Patient 1

**Fig. 1:**
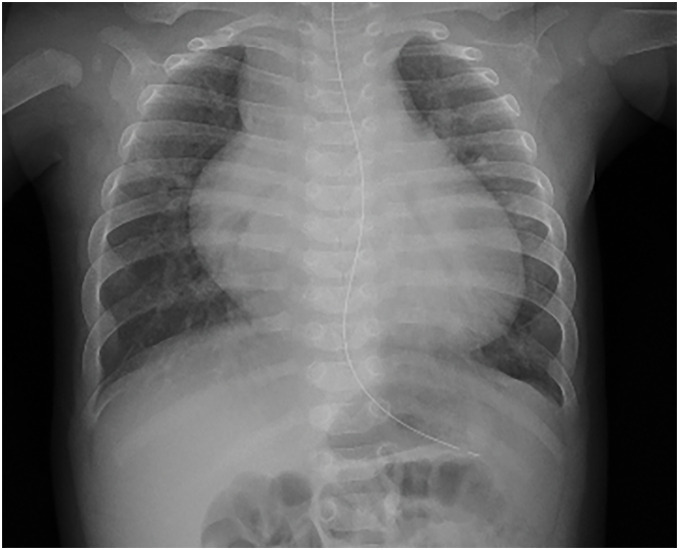
Preoperative chest radiograph of patient 1. There is significant cardiomegaly.

**Fig. 2:**
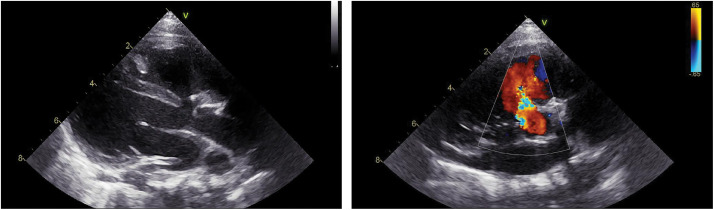
Preoperative transthoracic echocardiogram of patient 1. The echocardiogram shows a perimembranous VSD and dilated LV and RV. LV, left ventricle; RV, right ventricle; VSD, ventricular septal defect

The patient was a 7-month-old boy, weighing 6.9 kg, who was prenatally diagnosed with a VSD. There was no family history of genetic disorders. He presented with dysmorphic features such as short stature, hypertelorism, a saddle nose, and hyperextensible skin. Genetic testing revealed Klinefelter syndrome (47, XXY) and a *TAB2* gene variant. Although cardiac function was preserved after birth, he developed congestive heart failure with impaired cardiac function during early infancy (**Fig. 1**). Echocardiography at 4 months of age demonstrated a large perimembranous VSD (**Fig. 2**), a reduced LVEF of 46%, and an LV end-diastolic diameter of 31 mm (126% of normal). Preoperative cardiac catheterization showed severe pulmonary hypertension (90% of systemic blood pressure), a pulmonary-to-systemic flow ratio (Qp/Qs) of 2.2, and an indexed pulmonary vascular resistance of 4.7 WU·m^2^. Both LV and RV end-diastolic volumes were significantly enlarged (277% and 279% of normal, respectively), while LVEF and RVEF were decreased (48% and 38%, respectively). Despite optimal medical management, his heart failure worsened, necessitating surgical VSD repair. Using cardiopulmonary bypass with aortic and bicaval cannulation, mild hypothermia, and aortic clamping with antegrade myocardial protection with cold blood cardioplegia, the VSD was closed with a patch. Postoperatively, he experienced low cardiac output and persistent pulmonary hypertension, requiring significant inotropic support and inhaled nitric oxide. He was successfully extubated on POD 7 and was managed with medications including carvedilol, enalapril maleate, and sildenafil citrate. Two years after surgery, the patient remains in New York Heart Association (NYHA) functional class I, although developmental delay remains present. Recent echocardiography demonstrated an improved LVEF of 67%, an improved LV end-diastolic diameter of 26 mm (90% of normal), and mild elevation of tricuspid regurgitation flows, suggesting mild pulmonary hypertension.

### Patient 2

**Fig. 3:**
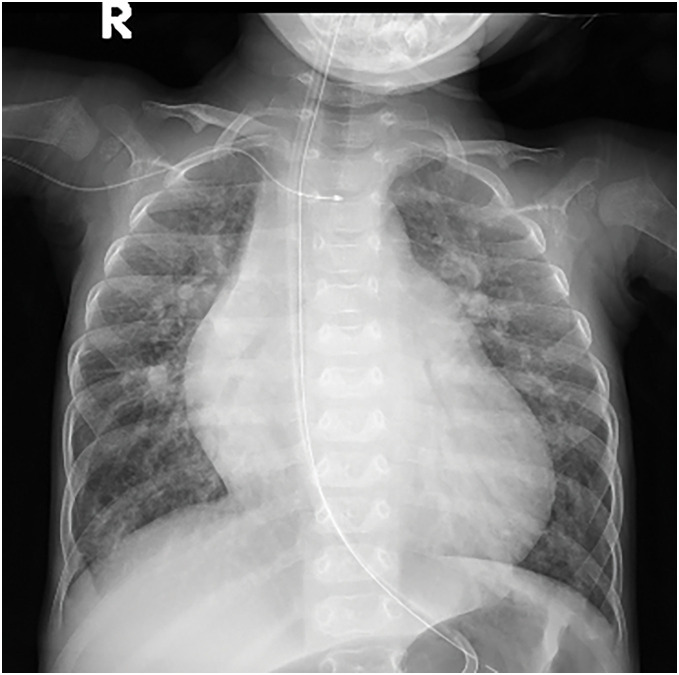
Preoperative chest radiograph of patient 2. There is significant cardiomegaly and pulmonary congestion.

**Fig. 4:**
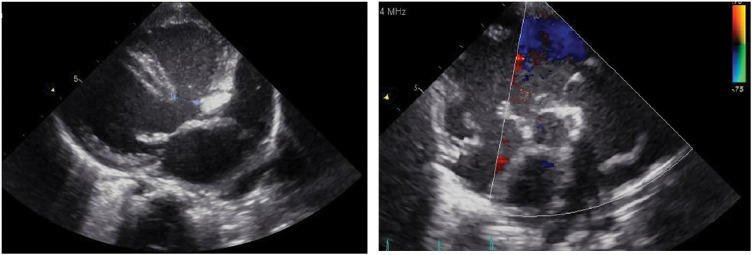
Preoperative transthoracic echocardiogram of patient 2. The echocardiogram shows a perimembranous VSD and dilated LV and RV. LV, left ventricle; RV, right ventricle; VSD, ventricular septal defect

The patient was a 1-year-4-month-old boy, weighing 5.4 kg, who was diagnosed with a VSD and reduced ventricular function (LVEF 50%) shortly after birth. No family history of genetic disorders was detected. He presented with extracardiac manifestations, including a broad forehead, saddle nose, micrognathia, low-set ears, and joint laxity. Chromosomal microarray analysis revealed a 1.2-Mb deletion in 6q24.3–q25.1, involving the *TAB2* gene. He developed congestive heart failure associated with VSD and underlying cardiomyopathy (**Fig. 3**). Due to decreased biventricular function, treatment with carvedilol and enalapril maleate was initiated at 3 months of age. He was referred to our hospital for further treatment at 8 months of age. His LVEF and RVEF gradually improved from 45% to 70%, and from 18% to 51%, respectively, by 1 year of age following intensified medical therapy, although LV end-diastolic diameter remained 33 mm (135% of normal). Echocardiography demonstrated a perimembranous VSD (**Fig. 4**). Preoperative cardiac catheterization showed pulmonary hypertension (60% of systemic blood pressure), Qp/Qs of 2.5, and an indexed pulmonary vascular resistance of 2.0 WU·m^2^. Both LV and RV end-diastolic volumes were enlarged (226% and 155% of normal, respectively), while LVEF and RVEF were preserved (63% and 62%, respectively). However, 2 days after catheterization, he suffered a high-grade fever and cardiogenic shock due to acute congestive heart failure, necessitating emergent initiation of ECMO. ECMO was successfully weaned after 5 days, but inotropic support (dobutamine and milrinone) was continued to maintain his ventricular function. Although heart transplantation or a ventricular assist device was not pursued by his family, they requested surgical VSD repair. Cardiopulmonary bypass commenced with aortic and bicaval cannulation using mild hypothermia. After the ascending aorta was clamped after infusion of cold blood cardioplegia, the VSD was closed with a patch. Although weaning from bypass was initially successful, he developed postoperative biventricular failure (LVEF 10% and RVEF 10%) and hypotension despite escalation to high-dose epinephrine and nitric oxide, requiring emergent ECMO via central cannulation 4 days after surgery. ECMO was decannulated after 7 days, followed by delayed sternal closure 1 week later. Subsequently, he developed mediastinitis caused by *Pseudomonas aeruginosa*, which required mediastinal irrigation and primary chest closure 25 days after the initial VSD repair. He was eventually extubated on POD 45, and medical therapy with carvedilol and enalapril maleate was resumed. One year and 3 months after surgery, the patient remains in NYHA functional class I, though he has a developmental delay. Recent echocardiography showed an LVEF of 67%, an improved LV end-diastolic diameter of 26 mm (88% of normal), and no evidence of pulmonary hypertension.

## DISCUSSION

To date, 84 patients with *TAB2* gene abnormalities, including both deletions and sequence variants, have been reported.^1–5)^ These patients frequently exhibit CHD, cardiomyopathy, and dysmorphic facial features such as frontal bossing, hypertelorism, ptosis, low-set ears, and dental problems. Additional clinical findings include short stature, connective tissue abnormalities, hypotonia, hearing loss, and developmental delay.^1–5)^ We report 2 additional children with *de novo TAB2* gene abnormalities who exhibited similar phenotypes. These findings suggest that genetic workup should be considered for patients presenting with the combination of CHD, cardiomyopathy, and the phenotypic features identified in this study.^1–5)^

In human embryos, *TAB2* is expressed in endothelial cells of the ventricular trabeculae and the endocardial cushions. As the endocardial cushions are essential for heart valve formation, valvular abnormalities represent the most prevalent cardiac manifestation in the *TAB2*-related phenotype. Mitral valve defects occur most frequently, often in combination with other valvular lesions. Although most CHD cases associated with *TAB2* abnormalities are asymptomatic, severe defects such as hypoplastic left heart syndrome have been reported in 2 patients. Other common defects include atrial septal defects and VSD. Surgical interventions for cardiac disease have been reported in 14 patients (17%), including a limited number of successful VSD repairs.^1–5)^ However, there have been no detailed reports specifically addressing VSD repair in children with comorbid cardiomyopathy associated with *TAB2* gene abnormalities.

Another significant cardiac concern in patients with *TAB2* gene abnormalities is cardiomyopathy associated with impaired ventricular function.^1–5)^ MAP3K7 regulates cardiomyocyte homeostasis by inducing either pro-survival or cell death signaling pathways.^2,3,6)^ When MAP3K7 signaling is diminished due to *TAB2* deficiency, the balance shifts toward cell death, resulting in cardiomyopathy and cardiac dysfunction.^2,3,6)^ This suggests that *TAB2* is not only critical for cardiac morphogenesis but also plays a vital role in maintaining primary myocardial function.^3)^ The clinical presentation of *TAB2*-related cardiomyopathy is highly variable.^1–6)^ While some children succumb to early-onset systolic heart failure, other patients do not develop cardiomyopathy until adulthood.^1–6)^ In our cases, both children developed cardiac dysfunction from early infancy that was disproportionate to the severity of the VSD shunts and therefore were diagnosed with concomitant cardiomyopathy. Although they successfully tolerated VSD repair, their clinical courses suggest that families should be informed that postoperative cardiac dysfunction may occur, requiring mechanical support. Moreover, these patients are scheduled for ongoing cardiological follow-up, including regular echocardiography to monitor ventricular function and valvular integrity even after surgical recovery.

Approximately one-third of infants experience LV dysfunction following VSD repair; however, this dysfunction typically resolves in most patients within 3 months and generally by the 9-month follow-up.^7,8)^ Surgical VSD closure induces myocardial injury and a systemic inflammatory response, both of which contribute to postoperative LV dysfunction.^7,8)^ Furthermore, VSD closure acutely increases LV afterload, as the ventricle must transition from pumping into the low-resistance pulmonary vascular bed to the higher-resistance systemic circulation.^8)^ A reduction in LV preload following VSD closure may also exacerbate postoperative dysfunction, particularly in patients with significant preoperative LV dilation.^7,8)^ In our report, both children presented with extensive LV dilation due to large VSDs and comorbid cardiomyopathy, subsequently experiencing severe postoperative ventricular dysfunction. Notably, the older patient suffered a cardiovascular collapse requiring ECMO support. These cases suggest that earlier VSD repair should be considered—before the development of extensive LV dilation—to mitigate the risk of severe postoperative dysfunction in children with *TAB2*-related cardiomyopathy. Furthermore, the favorable clinical recovery observed in our patients suggests that surgical VSD repair is justified and may improve long-term outcomes in children with *TAB2* gene abnormalities.

Finally, multidisciplinary genetic counseling should be provided to their families. In our cases, genetic counseling facilitated a deeper parental understanding of CHD and the underlying cardiomyopathy, reinforcing the need for lifelong cardiac surveillance.

## CONCLUSIONS

In conclusion, successful VSD closure was achieved in 2 children with dilated ventricles and impaired cardiac function associated with *TAB2* gene abnormalities. Although long-term follow-up remains essential, VSD repair may improve clinical outcomes in patients with *TAB2* gene abnormalities complicated by cardiomyopathy.

## DECLARATIONS

### Availability of data and materials

All data generated or analyzed during this study are included in this published article.
